# The Current Landscape of mRNA Vaccines Against Viruses and Cancer–A Mini Review

**DOI:** 10.3389/fimmu.2022.885371

**Published:** 2022-05-06

**Authors:** Reese Jalal Ladak, Alexander J. He, Yu-Hsun Huang, Yu Ding

**Affiliations:** ^1^ Rosalind and Morris Goodman Cancer Institute, McGill University, Montreal, QC, Canada; ^2^ Department of Biochemistry, McGill University, Montreal, QC, Canada; ^3^ Kennedy Institute of Rheumatology, Nuffield Department of Orthopaedics, Rheumatology and Musculoskeletal Sciences, Oxford University, Oxford, United Kingdom; ^4^ Department of Anatomy and Cell Biology, McGill University, Montreal, QC, Canada

**Keywords:** cancer vaccination, mRNA vaccine delivery, viral vaccination, mRNA vaccine developers, clinical trials of mRNA vaccines, mRNA vaccine mechanism, mRNA modifications

## Abstract

Both infectious viral diseases and cancer have historically been some of the most common causes of death worldwide. The COVID-19 pandemic is a decidedly relevant example of the former. Despite progress having been made over past decades, new and improved techniques are still needed to address the limitations faced by current treatment standards, with mRNA-based therapy emerging as a promising solution. Highly flexible, scalable and cost-effective, mRNA therapy is proving to be a compelling vaccine platform against viruses. Likewise, mRNA vaccines show similar promise against cancer as a platform capable of encoding multiple antigens for a diverse array of cancers, including those that are patient specific as a novel form of personalized medicine. In this review, the molecular mechanisms, biotechnological aspects, and clinical developments of mRNA vaccines against viral infections and cancer are discussed to provide an informative update on the current state of mRNA therapy research.

## Introduction

Since its establishment as a treatable disease in modern medicine, cancer is the second leading cause of death in the United States (1). In Canada, cancer has a 50% incidence and 25% mortality rate^1^ (2). Globally, respiratory cancers are the sixth most prevalent non-communicable causes of death^2^ (3). From a biopsychosocial perspective, cancer patients and families experience poor mental health outcomes and heavy financial burden (4, 5). Despite there being substantial improvements to traditional cancer treatments and developments of therapeutic strategies such as adoptive T cell immunotherapy and oncolytic viral therapy, these new treatments have their own shortcomings. For example, while effective against bloodborne cancers, T cell immunotherapy has limited efficacy against solid tumour cancers due to dosage restrictions imposed by off-target adverse effects of the T cells on healthy tissue (6) For oncolytic viral therapy, the technology still faces the primary challenge of finding the optimal level of immunogenicity the virus should possess, such that a robust antitumour immune response is induced but not insofar as to compromise the virus reaching its target site (7). Hence, there is still a need for the development of novel cancer treatments.

Infectious viral diseases have been a persisting epidemiological and clinical issue, both before and after the advent of vaccines. At the time of writing, Severe Acute Respiratory Syndrome Coronavirus-2 (SARS-CoV-2) has infected over 480 million people and caused six million deaths worldwide^3^ (8). Yet even before the COVID-19 pandemic, the seasonal flu has consistently caused over 15 million symptomatic infections, 100,000 hospitalizations annually in the past decade in the US alone, and 294,000-518,000 global annual respiratory deaths^4^ (9, 10). Additionally, viruses effectively managed by vaccines and well-established treatment standards in developed countries still pose a significant burden of disease in the developing world, where Human Immunodeficiency Virus (HIV), Rabies, Ebola and Influenza are rampant (11–14). For these reasons, additional vaccine technologies are crucial for improving the global health burden of viral diseases.

The ability to administer mRNA engineered *in vitro* that later translates into an antigen (Ag), bypasses the need to use vaccination platforms that risk harmful effects such as live-attenuated viruses that could revert to more virulent forms, or ones that require large biological investments such as protein-based platforms which typically require adjuvant supplementation for an adequate immune response (15, 16). Although previously neglected due to its instability, mRNA has now regained focus following advancements in their synthesis, delivery, and immunogenicity-optimizing and stabilizing techniques (17–20). Further, with their scalability, cost-effectiveness and adaptability, mRNA vaccines are emerging as a promising therapeutic strategy for both viral diseases and cancer (21, 22). The first report of successful mRNA delivery into an *in vivo* model was done in 1990, where protein levels of chloramphenicol acetyltransferase, luciferase and β-galactosidase were successfully detected upon injection of the respective mRNAs into the skeletal muscle of mice (23). Later, the therapeutic use of mRNAs was exemplified *via* the first *in vivo* study of a liposome-encapsulated mRNA as a vaccine. The similarity of immunoprotective outcomes between mouse models treated with the mRNA-based vaccine and those challenged with Influenza highlighted the efficacy of the mRNA vaccine platform (24). In this review, we explore mRNA vaccine mechanisms, biotechnology, and clinical trials against select viral infections and cancer to highlight the excellent prospects of this emerging next-generation vaccination platform.

## mRNA Vaccine Mechanism and Routes of Administration

**Figure 1:**
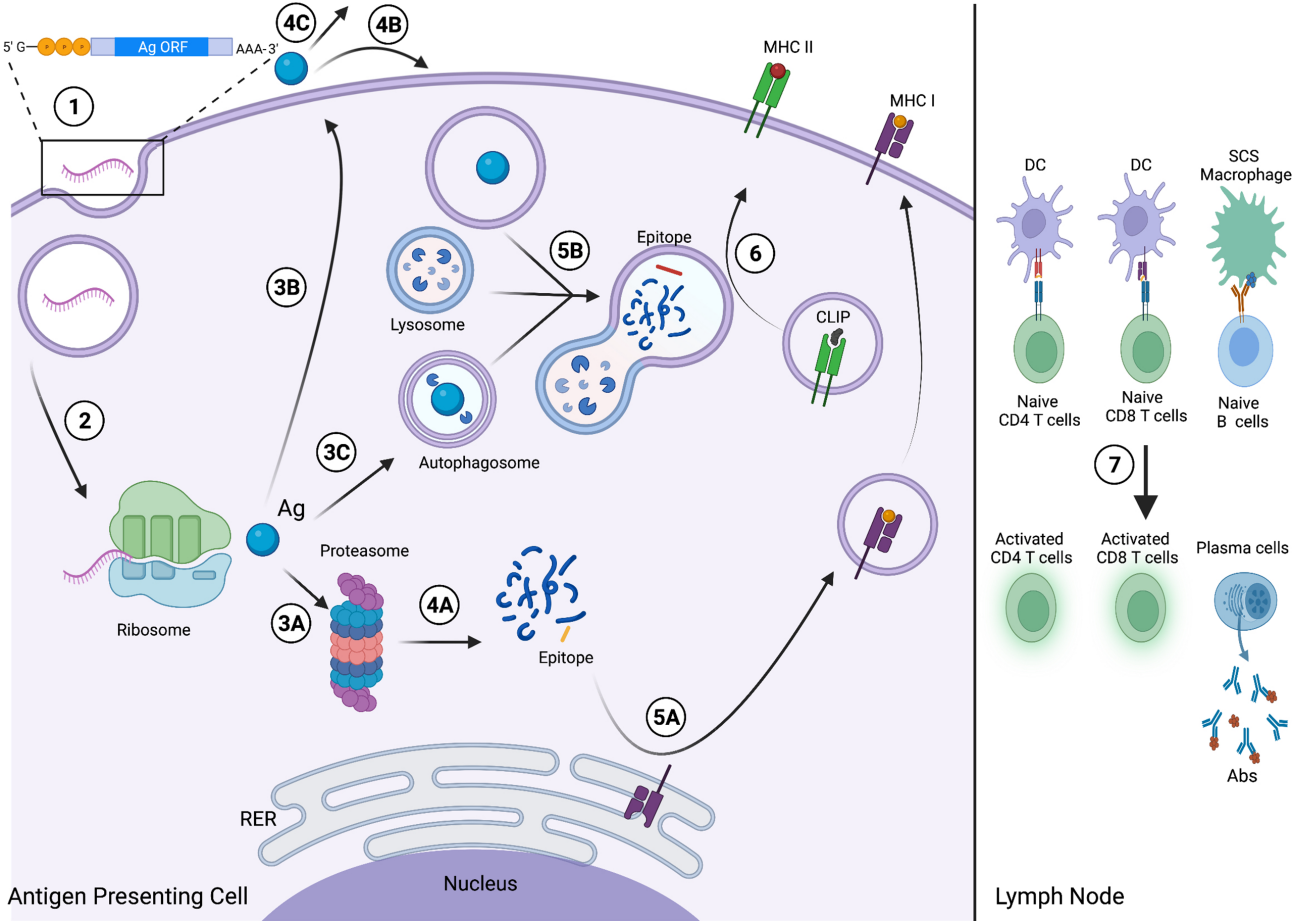
Viral and Cancer RNA vaccine mechanism. (1) The viral Ag, TAA or TSA-encoding mRNA enters the APC and then the cytosol following endosomal escape. (2) The mRNA is translated by the host ribosomal machinery into the encoded Ag, which is then either (3A) degraded by the proteasome and enters the endogenous MHC I pathway; (3B) exocytosed from the APC *via* the secretory pathway; or (3C) enters the autophagic pathway and is internalized by an autophagosome. (4A) Proteasome-mediated degradation yields fragments (blue), including the epitope (yellow). Following secretion, the Ag either (4B) enters an APC *via* endocytosis and then the exogenous MHC II pathway; or (4C) the native antigen will circulate through the lymphatic system to secondary lymphoid organs. (5A) The epitope binds MHC I at the rough endoplasmic reticulum (RER) and traffics to the cell membrane for surface presentation *via* the secretory pathway. (5B) The Ag, internalized *via* endocytosis or processed by autophagy, is digested into fragments, including the epitope (red), *via* lysosome-mediated degradation. (6) The epitope binds MHC II and displaces class-II associated invariant chain peptide (CLIP), and the complex then traffics to the membrane for surface presentation. (7) The APCs migrate to secondary lymphoid organs, including the lymph node. The APCs present the epitope-loaded MHC I and MHC II complexes to activate naive CD8 and CD4 T cells, respectively, and follicular DCs and subcapsular sinus (SCS) macrophages present the native Ag to activate naive B cells, driving plasma cell differentiation and Ab production. This figure was created with BioRender.com.

RNA vaccine technology harnesses the simplicity of the central dogma of molecular biology (23, 25). Upon entering antigen-presenting cells (APCs), the mRNA is translated by ribosomes to produce the encoded Ag of interest to then activate humoral (antibody-dependent) and/or cell-mediated immunity (26). Humoral immunity is activated when the Ag is secreted in its native state. Upon arriving at the lymph node, the Ag is presented on the surface of follicular dendritic cells (DCs) and subcapsular macrophages to cognate B cell receptors (BCRs), thereby activating and differentiating B cells into plasma cells that produce Ag-specific antibodies (27–29). While humoral immunity is activated by recognition of the epitope on the native Ag, cell-mediated immunity relies on recognizing the epitope loaded on Major Histocompatibility Complex (MHC) molecules. Following translation, the APC can process and present the Ag on MHC class I (MHC-I) and MHC class II (MHC-II) to interact with CD8 or CD4 T cells, respectively, to activate T cell-mediated immunity. Finally, the primary cell-mediated and humoral immune responses elicited by mRNA vaccines result in the proliferation of memory T and B cells, which offer long-term protection against secondary immune responses. This mechanism is summarized in **Figure 1**.

Injection is the preferred route of mRNA vaccine administration. Currently, intramuscular (IM) is the most common means of local delivery and is particularly common for DC-independent mRNA vaccines (30). The rich vasculature of muscular tissue enables efficient re-circulation and movement of Ags and APCs to activate adaptive immune cells, making IM injections desirably immunogenic (30). The intradermal (ID) route is another promising route of administration, as the dense vascularity and extensive lymphatic drainage of the dermis provides an ideal microenvironment for efficient Ag trafficking by APCs (31). Subcutaneous (SC) injection is also a possible means of local delivery since the loose adipose tissue of the subcutis can sustain large injection volumes, and cause less pain (32, 33). Additionally, intravenous (IV) administration may also be employed to deliver the vaccine systemically, and permits the largest volume of administration compared to other routes (34). In the case of cancer treatment, intratumoural (IT) injections are also being explored (35, 36). Finally intranodal (IN) delivery is another route, which poses an efficient means of rapid engulfment of the mRNA payload by APCs (37–39).

## mRNA Vaccine Biotechnological Developments

### Synthesis Principles and Encoded Antigen Targets

mRNA synthesis uses cell-free, *in vitro* transcription (IVT)-based platforms, where a DNA plasmid encoding the desired full-length sequence is transcribed (23). This is advantageous compared to cell-based platforms as IVT provides increased control over mRNA production and chemical modifications to improve its stability, bypassing this historical limitation of mRNA (40). In addition, these modifications served to optimize mRNA immunogenicity to elicit a robust immune response, and to bypass innate immune sensors (i.e. Pattern Recognition Receptors) from driving its premature degradation.

These modifications include 5’-cap, 3’Poly(A)-tail and untranslated region (UTR) alterations, which are important regulators of mRNA translation (41). Reviewed in (21, 42), 5’ cap modification strategies include designing anti-reverse synthetic cap analogs and adopting the Vaccinia Virus capping enzyme (43, 44). Today, however, transcripts are commonly co-transcriptionally capped using CleanCap developed by TriLink BioTechnologies (45). Biotechnological approaches to improving mRNA half-life *via* the 3’-Poly(A) tail involve extending it by encoding more adenosine bases in the DNA template or by enzymatically adding canonical or modified nucleobases to the Poly(A) tail of the mRNA (46).

Stability and translation of mRNAs can also be improved by encoding the 5’ and 3’ UTRs with different regulatory sequences, derived from viral or eukaryotic origins (42, 46–48). Amongst these sequences are the 3’ UTR of the eukaryotic elongation factor 1α and Orthopoxvirus Virus-derived 5’ UTR sequences that prevent decapping and degradation (42). Other modifications include incorporating modified nucleobases such as pseudouridine and 5-methylcytidine, which have been shown to improve mRNA translation efficiency (49). Another modification option is editing the codon region itself but optimizing the codon region may disrupt potent cryptic epitope-mediated T cell activation, compromising vaccine-induced immunity (50, 51).

The mRNA itself encodes an Ag which elicits effective humoral and T cell-mediated immunity when targeted by the immune system *in vivo*. For viral vaccines, the Ag is often a structural protein required for receptor-binding and/or fusion. Such mRNA vaccines against viruses often depend on epitope accessibility and mutation rate. For example, HIV-1 vaccine development is limited by the highly glycosylated structure of the envelope protein (Env) that hinders the binding of neutralizing antibodies (52–56). Env also exhibits high antigenic diversity both between and within infected individuals due to the high mutation rate of HIV-1 (57, 58). Consequently, both of these factors drive the immune escape of HIV-1 and should also be factors to consider when deciding on an Ag target for other viral mRNA vaccines.

In cancer, the target is commonly a tumour-associated antigen (TAA) or a tumour-specific antigen (TSA). In cancer cells, TAAs are antigenic proteins that are overexpressed, while TSAs are proteins that are uniquely expressed (59). Due to the presence of TAAs on both healthy and cancerous cells, mRNA vaccines encoding TAAs have presented limited efficacy due to T cell tolerance (60, 61). By contrast, because TSAs (ie. neoantigens) arise from extensive mutations in cancerous cells and are considered foreign by the immune system, the peptide/MHC affinity of T cell-bearing TCRs is greater when TSA epitopes, rather than TAA epitopes, are loaded, resulting in a more robust immune response (62–64).

### Delivery Strategies

Immunogenicity has been observed with direct inoculation of naked mRNA, as seen in the first naked mRNA vaccine clinical trial by Weide et al. in 2008, where successful injections of autologous mRNA in melanoma patients demonstrated its safety and feasibility. Additionally, an anti-tumour humoral immune response was also elicited in some patients, but clinical regression was not observed (65). These observations could be attributed to the fact that mRNA is often unstable *in vivo*, undergoing degradation by extracellular RNases before they can be internalized (66).

To address mRNA stability, delivery strategies include encapsulating mRNA within lipid nanoparticles (LNPs), DCs or DC extracellular vesicles (DEVs). LNPs are now widely used as a vehicle for mRNA delivery, and initial iterations were composed of cationic amphipathic lipids that strongly bound the negatively charged mRNA to protect it during delivery. The first major breakthroughs typifying the use of cationic lipids came from an *in vitro* study by Malone et al., when he successfully transfected *Photinus pyralis* Luciferase mRNA into human and other animal cells using cationic liposomes as a delivery system (67). *In vivo* mouse model studies more recently performed by Kranz et al. in 2016, demonstrated that Ag-encoding mRNAs in complex with liposomes (RNA-LPX) induce robust effector and memory T cell immunity, and tumour rejection. This was due to the liposome facilitating efficient DC uptake upon injection. Additionally, they also initiated a Phase I trial using their RNA-LPX technology, the first ever clinical trial of an mRNA-encapsulated liposome vaccine, a milestone that set the precedent for several following studies using liposome-encapsulated RNA vaccines (68). While they greatly improve mRNA stability, LNPs have been reported to still trigger damaging immunostimulation and oxidative stress (69, 70). As such, newer LNP technologies use ionizable lipids, which aid in improved mRNA delivery and safety. Upon injection, these LNPs are neutral under the physiological pH of the bloodstream, thereby mitigating toxicity and prolonging circulation time compared to cationic LNPs (71). Following endocytosis, the LNP polarizes in the acidic endosome, driving the release of the mRNA into the cytosol for translation (46). The first case where ionizable LNPs were demonstrated to be an effective alternative lipid-based delivery method in the 2006 landmark experiment by Zimmerman et al. that used this system to deliver apolipoprotein B (ApoB)-specific siRNA into hepatocytes (72). Following intravenous administration, successful silencing of ApoB was observed.

mRNA virus and cancer vaccine delivery can also be accomplished by loading DCs, which is accomplished either *via* an *ex vivo* or *in situ* approach. The establishment of DCs as a vehicle for mRNA vaccine therapeutics can be attributed to a study done by Boczwoski et al. in 1996, where *in vivo* mouse models that received an mRNA-loaded DC vaccine were adequately protected from subsequent tumour challenge, and immunocompromised mouse models immunized with the vaccine were shown to have substantially fewer metastases (73). In 1996, Hsu et al. of the Stanford University Medical Center performed the first DC-based mRNA vaccine clinical trial. Upon injecting the *ex vivo*-loaded vaccines, all four B cell lymphoma patient participants developed detectable antitumour responses with one patient undergoing complete tumour regression. These results signified a major breakthrough in the development of DC-based mRNA vaccines (72). In the *ex vivo* approach, autologous DCs are transfected with the mRNA encoding the Ag and co-stimulatory molecules through electroporation to drive differentiation and activation. Upon reinfusion, the DCs present the encoded Ag to activate lymphocytes. In the *in situ* approach, the mRNA vaccine is injected directly into the lymph nodes where they enter into DCs and present the Ag. This technique is exemplified by experiments that intranodally injected TAA-encoding and immunomodulating TriMix mRNA intranodally in several different cancer mouse models, which led to marked Ag-specific T cell stimulation and CTL antitumour activity (74). Although promising, one concern regarding DC-based vaccines is that while large numbers of DCs are required for effective treatment, while there is only a limited number of naturally circulating DCs.

Extracellular vesicles (EVs), derived from various cell populations, including dendritic cells and tumour cells, or synthesized artificially, have exhibited some promising cancer and viral vaccine applications (75–79). DEVs are vesicles formed and secreted by DCs, either *via* direct budding or within exosomes, to act in an autocrine, paracrine or endocrine manner (80). DEVs contain co-stimulatory molecules, cell adhesion molecules, immune cell-activating ligands, and MHC I- or MHC II-loaded TAAs/TSAs or viral antigens to activate lymphocytes (76). Additionally, DEVs can also express TNF super family ligands, hence allowing them to facilitate the direct killing of tumours and activation of NK cells (81). Therapeutically, DEVs loaded with antigenic peptides and full antigens have shown significant efficiency at activating T cells, reducing tumour size and increasing overall survivability in animal models (76, 80). In terms of viral diseases, recent research has demonstrated that administration of DEVs presenting Respiratory Syncytial Virus (RSV) M, L and NS proteins elicited a robust Ag-specific CD8+ T cell response in mice (82). Their therapeutic effects towards cancer and viral disease when loaded with mRNA have not yet been explored, but preliminary studies have suggested that DEVs will excel at rapid delivery of vaccine materials (83). There are several advantages of DEVs over traditional DC-based mRNA vaccines, reviewed in (80). Firstly, the molecular composition of DEVs is more controllable than those of whole-cell systems. Secondly, compared to DCs, DEVs have a longer shelf-life, allowing them to systemically persist for longer and induce more robust immune responses. DEVs can also be engineered to target to secondary lymphoid organs independent of any chemotactic factors, unlike DCs that depend on chemokine-mediated migration. Lastly, DEVs transfer its Ag-MHC complex onto different host DC subpopulations, increasing the number of DCs carrying this specific Ag and thus amplifying T cell activation in the downstream adaptive immune response (84).

In addition to the use of LNPs and DCs, there exists other strategies of mRNA delivery, including the use of polymer-, peptide-, and squalene-based systems. Cationic polymers are capable of forming structurally heterogeneous complexes with mRNA called polyplexes, which can be endocytosed by cells to ultimately deliver mRNA into the cytosol through an unclear endosomal escape mechanism. Polyethylenimine is currently the most well-studied polymer used for this purpose, but faces challenges around its cytotoxicity despite offering compelling transfection efficiency (85). Poly(beta-amino ester)s, poly(amidoamine)s and ionizable aminoethylene-conjugated poly(aspartamide)s are other polymers being investigated as polyplex delivery systems. Cationic or amphipathic peptide systems are another alternative for delivery. Peptides with arginine-alanine-leucine-arginine (RALA) motifs effectively form nanocomplexes with mRNA and facilitate subsequent mRNA endosome escape in a pH-dependent manner. This method is exemplified when this delivery system was used to successfully transfect DCs *in vitro*, and induce T cell-mediated immunity *in vivo* (86). In addition, PepFect14 is another cell-penetrating peptide which can transfect mRNA *in vivo*, including in ovarian cancer xenograft models and insofar as outperforming lipid-mediated transfection (87). Protamine is another important peptide serving not only as an mRNA delivery agent, but also as a potential vaccine adjuvant due to its marked immunostimulatory properties when complexed with mRNA (35, 88–90). Protamine-protected mRNA was also the first non-lipid nanocomplex mRNA vaccine to undergo clinical trials (91). Lastly, squalene-based cationic nanoemulsions to deliver mRNA are also emerging as a promising delivery system. Structurally, this is formulated by a squalene core with a lipid shell onto which the mRNA payload is localized, and was shown to elicit remarkable antibody and T-cell responses when introduced into primates (92).

## Clinical Trials of Viral mRNA Vaccines

While measures such as social distancing and mask mandates slowed COVID-19 transmission, vaccines continue to be an essential means of reducing morbidity and mortality in the pandemic (93). Current mRNA vaccines are designed against the viral spike (S) protein, encoding it either as a transmembrane full-length protein or secreted receptor binding domain (RBD). The Pfizer-BioNTech BNT162b2 and Moderna mRNA-1273 vaccines are the only approved mRNA vaccine candidates, conferring 95% and 94.1% vaccine efficacy, respectively, in Phase III trials (94, 95). Although their efficacies have waned since their introduction, full vaccination continues to protect against hospitalization and death, especially with recent variants, with a booster dose enhancing this outcome (96–100). Several other mRNA vaccines against COVID-19 are also in development. Phase I trials of the nucleoside-unmodified, S protein RBD-encoding mRNA vaccine candidate ARCoV reported remarkable levels of anti-RBD and neutralizing antibodies 7 to 14 days after the second dose, and an elevated T cell-mediated immune response 14 to 28 days after (101). Interestingly, administering 15μg of the vaccine elicited higher levels of neutralizing antibodies compared to those of naturally infected patients. In addition, CVnCoV is also in ongoing Phase III trials, preliminarily reporting a 70.7% vaccine efficacy against moderate-to-severe disease with an acceptable safety profile (102).

The influenza virus Types A and B cause seasonal epidemics, and the mRNA platform is emerging as a flexible means of countering the antigenic drift of new strains. Currently, these vaccines encode the Haemagglutinin (HA) protein as the Ag target. There are multiple influenza mRNA vaccines in clinical trials pursued by Moderna, Pfizer, and Translate Bio/Sanofi. The Moderna mRNA-1011 candidate is an LNP-encapsulated, quadrivalent mRNA vaccine encoding for four seasonal outbreak influenza virus HAs and is currently leading Phase II trials. In Phase I, mRNA-1011 successfully yielded neutralizing antibody titers against all target influenza strains 29 days after vaccination at 50 µg, 100 µg and 200 µg doses in young (age 18–49) and old (age 50+) patient cohorts without severe side effects (103). In addition to these candidates, current research is also in the midst of using this platform to develop a universal influenza vaccine (104).

To date, there are no licensed vaccines targeting HIV-1. As previously mentioned, vaccine development is limited by its high mutation rate and antigenic variability that manifests both between and within patients as heterogeneous “quasi-species” (57, 58). mRNA, however, has the potential to become an efficacious vaccine platform because its IVT-based production system is rapidly adaptable to encode new Ags to counter this diversity. As mentioned, current mRNA vaccines target Env, and emerging data maintains this as a promising approach for next-generation vaccination strategies. Four mRNA vaccine candidates are currently in early clinical trials, with some promising results emerging. In particular, Moderna and the International AIDS Vaccine Initiative (IAVI) are developing an mRNA vaccine encoding the eOD-GT8 60mer, a 60-subunit, self-assembling nanoparticle targeting the Env outer domain (105). Preliminary phase I data from the Moderna-IAVI trials reported that after two priming doses with this candidate, 97% of participants developed IgG B cells, precursors to broadly neutralizing antibodies against HIV-1^5^ (106). Alternatively, some work had also been done to use mRNA to encode for broadly neutralizing antibodies against conserved receptor-binding regions on Env to confer passive immunity against HIV-1 (107).

mRNA vaccines against several other viruses are also being pursued by different groups. Zika virus, Rabies Virus, RSV, Chikungunya Virus, Human Metapneumovirus and Cyclomegalovirus all have mRNA vaccine candidates in clinical trials, with many developed by Moderna. A self-amplifying Ebola glycoprotein-encoding mRNA vaccine successfully elicited Ag-specific IgG production and T cell response (108). CureVac is also developing a capsid protein-encoding rotavirus mRNA vaccine (109). Taken together, mRNA is an invaluable tool for the development of next-generation viral vaccines that could be readily adjustable to accommodate the high genetic and antigenic variation generally seen in RNA viruses (110).

## Clinical Trials of Cancer mRNA Vaccines

mRNA vaccines are also currently being explored as a therapeutic intervention to cancer. Several pharmaceutical companies are hosting clinical trials for mRNA cancer vaccine candidates. BioNTech has the BNT111–115 candidates in clinical trials. Currently in Phase II, BNT111 is designed to treat advanced melanoma, and is administered in a LNP in combination with the cell cycle checkpoint inhibitor and PD-1 blocker Celiplimab. The BNT111 mRNA itself encodes four different TAAs (NY-ESO-1, MAGE-A3, tyrosinase, TPTE) and was shown, alone and in combination with Celiplimab, to be safe and to preliminarily improve survival and elicit a robust T cell response in phase I trials (111). Like BNT111, BNT113 is also in Phase II trials. Designed to treat Human Papillomavirus 16 (HPV16)-positive head and neck cancers, BNT113 encodes HPV16-derived neoantigens E6 and E7. Mouse model experiments have indicated that mRNA vaccines encoding E7 induce complete tumour regression, successfully prevent relapse, and trigger robust immune infiltration into the tumour microenvironment (112). Currently, BNT113 is being administered alone and in combination with the PD-1 blocker Pembrolizumab. There are several other BioNTech mRNA cancer vaccines being studied: BNT112 and BNT115 are candidates currently in Phase I designed to treat prostate and ovarian cancers, respectively.

Due to the flexibility of mRNA design, these vaccines can be tailored to treat patient-specific cancers. Both BioNTech and Moderna have invested in designing personalized vaccines. From a patient tumour sample, neoantigens are characterized through high-throughput sequencing and immunologic screening to evaluate the immunogenicity of each neoantigen candidate (113). In particular, BioNTech, in collaboration with GeneTech, has launched the individualized Neoantigen specific Immunotherapy (iNeST) platform, headlined by the BNT122 candidate designed for various local and metastatic solid tumours. After a successful Phase Ib trial in which significant neoantigen-specific immune responses were observed, BNT122 is now undergoing Phase II trials as a monotherapy and in combination with monoclonal PD-1/PD-L1 antibody blockers (114). Similarly, Moderna has designed mRNA-4650, a personalized vaccine that can encode up to 20 defined patient-specific antigens, that is being tested on melanoma, gastrointestinal and genitourinary cancer patients (113). mRNA-4157 is another personalized vaccine by Moderna that uses an LNP as the delivery vehicle. Currently, mRNA-4157 is in Phase II trials to treat head and neck squamous cell carcinoma following Phase I trial success, where it was shown that when administered in combination with anti-PD-1 antibody Pembrolizumab, this candidate was more effective than Pembrolizumab alone (115). Using bioinformatics and exome sequencing, Moderna is also exploring the potential of encoding driver gene mutations detected in autologous tumours such as p53 and KRAS. mRNA-5671 encodes mutated KRAS to treat colorectal, NSCLC and pancreatic cancers induced by one of four of the most common cancer-driving KRAS mutations (G12D, G12V, G13D and G12C) (116, 117). Currently, mRNA-5671 is in Phase I trials, in which its safety is being evaluated as a monotherapy and in combination with Pembrolizumab.

There are also several other platforms that BioNTech and Moderna are exploring. These include the cytokine-encoding mRNAs (BioNTech BNT 151–153 and Moderna mRNA 2752) that induce amplified T cell responses and overcome tumour-mediated immunosuppressive effects. Pre-clinical data demonstrated mRNAs encoding pro-inflammatory cytokines induced enhanced cytolytic CD8 T cell activity and rendered the tumours more sensitive to checkpoint inhibitor therapy. This led to delayed tumour growth independent of any encoded TAA or TSA (36, 118). BioNTech is also exploring bispecific monoclonal antibody-encoding mRNAs (i.e. BNT141), termed RiboMabs, which can propagate immune responses by recruiting immune cells to tumours. Clinical trials where such mRNA-encoded antibodies were directly administered showed higher success rates compared to those of chemotherapy (119). These platforms are currently in Phase I clinical trials.

In addition to BioNTech and Moderna, CureVac has several mRNA cancer vaccines in trial, including the CV9202 candidate that was shown to be safe when administered in combination with checkpoint inhibitor therapy against NSCLC (120). Additionally, CV8102, their new vaccine against melanoma and other cancers, is currently undergoing Phase I testing alone and in combination with anti-PD-1 therapy. CureVac is exploring RNActive vaccines, which elicit a four to five-fold increase in protein expression capacity compared to regular mRNA vaccines. RNActive mRNA is also complexed with protamine to produce a self-adjuvanting mRNA vaccine that induces a stronger immune response in a TLR7-dependent manner (121). Argos Therapeutics is another company that explored mRNA vaccines against cancer and infectious disease. They used a DC-based platform, into which they inserted the Ag-encoding mRNA into DCs *via* electroporation. In 2008, they reported that the combination of cytokine-induced DC maturation, followed by co-insertion of the Ag-encoding and CD40L-encoding mRNAs *via* electroporation, effectively induced elevated IL-12 expression and a robust inflammatory response with Ag-specific T cells that exhibited memory phenotype (122). They had several vaccines in trials that have all since terminated, including one in 2018 that was stopped in Phase III due to lack of efficacy. They have not initiated any new cancer vaccine trials as of the time of writing.

Other active clinical trials are being hosted by groups including the University of Campinas against acute myeloid leukemia, the University Hospital Erlangen against Melanoma, the Olso University Hospital against prostate and other cancers, and the Memorial Sloan Kettering Cancer Center against multiple myeloma (21). Beyond the aforementioned, there are numerous institutions studying mRNA vaccine efficacy against cancer. There is a notable number of clinical studies to treat melanoma, many of which use DC-based platforms. Refer to **Table S1** for a full list of completed and active mRNA vaccine clinical trials against viruses and cancers.

## Concluding Remarks

While the use of mRNA as a therapeutic strategy has been studied for several decades, it was the emergence of SARS-CoV-2 that renewed interest in its potential. Currently, varying types of general and personalized mRNA vaccines are being developed against both viral diseases and cancer in tandem with work in optimizing mRNA synthesis, delivery, stability, efficacy and safety standards. Having previously been neglected due to its perceived instability, RNA-based therapy now spearheads an exciting new chapter in vaccinology.

## Author Contributions

RJL and AJH conceived the topic of the manuscript. RJL, AJH and Y-HH wrote the initial draft of the manuscript. RJL, AJH and YD contributed to the revision of the manuscript. All authors reviewed and approved the final version of the submission version.

## Conflict of Interest

The authors declare that the research was conducted in the absence of any commercial or financial relationships that could be construed as a potential conflict of interest.

## Publisher’s Note

All claims expressed in this article are solely those of the authors and do not necessarily represent those of their affiliated organizations, or those of the publisher, the editors and the reviewers. Any product that may be evaluated in this article, or claim that may be made by its manufacturer, is not guaranteed or endorsed by the publisher.

## Footnotes

1 Society CC. Canadian Cancer Statistics: 2021: Government of Canada; 2021 [Available from: https://cdn.cancer.ca/-/media/files/research/cancer-statistics/2021-statistics/2021-pdf-en-final.pdf?rev=2b9d2be7a2d34c1dab6a01c6b0a6a32d&hash=01DE85401DBF0217F8B64F2B7DF43986&_ga=2.25716733.1141201067.1645494864-721516791.1645494864].

2 Organization WH. The Top 10 Causes of Death. [Available from: https://www.who.int/news-room/fact-sheets/detail/the-top-10-causes-of-death.

3 Medicine JHU. COVID-19 Dashboard: By the Center for Systems Science and Engineering (CSSE) at Johns Hopkins University (JHU): John Hopkins; 2022 [updated 2022-02-21. Available from: https://coronavirus.jhu.edu/map.html.

4 Prevention CfDCa. Disease Burden of Flu [updated 2022-01-07. Available from: https://www.cdc.gov/flu/about/burden/index.html.

5 Venkatesan P. Preliminary phase 1 results from an HIV vaccine candidate trial. The Lancelet: Microbe2021 [Available from: https://www.thelancet.com/journals/lanmic/article/PIIS2666-5247(21)00042-2/fulltext.
